# Bibliometric analysis of intestinal microbiota and lung diseases

**DOI:** 10.3389/fcimb.2024.1347110

**Published:** 2024-02-15

**Authors:** Weiting Sun, Tong Zhou, Peng Ding, Liuxue Guo, Xiujuan Zhou, Kunlan Long

**Affiliations:** ^1^ Department of Critical Care Medicine, Hospital of Chengdu University of Traditional Chinese Medicine, Chengdu, China; ^2^ School of Clinical Medicine, Chengdu University of Traditional Chinese Medicine, Chengdu, China

**Keywords:** intestinal microbiota, lung, bibliometric analysis, VOSviewer, Citespace

## Abstract

**Background:**

Increasing evidence suggests a close association between the intestinal microbiome and the respiratory system, drawing attention to studying the gut-lung axis. This research employs bibliometric methods to conduct a visual analysis of literature in the field of intestinal microbiota and lung diseases over the past two decades. It offers scientific foundations for research directions and critical issues in this field.

**Methods:**

We retrieved all articles on intestinal microbiota and lung diseases from the SCI-Expanded of WoSCC on October 25, 2023. The analysis included original articles and reviews published in English from 2011 to 2023. We utilized Python, VOSviewer, and CiteSpace to analyze the retrieved data visually.

**Results:**

A total of 794 publications were analyzed. China ranked first in the number of publications, while the United States had the highest citations and H-index. Jian Wang was the most prolific author. Zhejiang University was the institution with the highest number of publications. *Frontiers in Microbiology* was the journal with the most publications. Author keywords appearing more than 100 times included “intestinal microbiota/microbiome”, “microbiota/microbiome”, and “gut-lung axis”.

**Conclusion:**

The correlation and underlying mechanisms between intestinal microbiota and lung diseases, including asthma, COPD, lung cancer, and respiratory infections, remain hot topics in research. However, understanding the mechanisms involving the gut-lung axis is still in its infancy and requires further elucidation.

## 1 Introduction

The intestinal microbiota refers to the microbial ecosystem within the human gastrointestinal tract, encompassing bacteria, fungi, viruses, and other microorganisms. The gene pool of this microbiota exceeds that of the human genome by a hundredfold (O’Hara and Shanahan, 2006; Grice and Segre, 2012; Ley et al., 2006). Changes in the composition and diversity of these microorganisms can impact not only the colonized organ but also distant organs and systems (Feng et al., 2018). The intestinal microbiota is closely linked to human health and is associated with various diseases, such as gastrointestinal disorders, metabolic diseases, and immune-related illnesses (Zhou et al., 2021; de Steenhuijsen Piters et al., 2015; Clemente et al., 2012). Current research indicates that dysbiosis of the intestinal microbiota is a fundamental cause of numerous gastrointestinal and non-gastrointestinal diseases and a range of mental health conditions (Carding et al., 2015; Forsythe et al., 2016). Recent evidence suggests intestinal microbiota is intricately connected to the respiratory system, acting as a critical regulatory factor in developing lung diseases like pneumonia, asthma, and chronic obstructive pulmonary disease (COPD) (Wang et al., 2017). While research on the gut-lung axis is still in its early stages, it holds potential as a novel approach for treating pulmonary diseases (Feng et al., 2018).

Bibliometrics is a quantitative research method used to analyze and assess scientific literature’s volume, quality, distribution, and impact. It involves statistical analysis of citations, author collaborations, and journal impact factors (Ma et al., 2021). Employing bibliometric methods can reveal the latest scientific research trends, hot topics, and interdisciplinary fields, as well as evaluate the impact and quality of academic achievements, providing decision support for the advancement of scientific research (Guler et al., 2016). No study has systematically explored the interrelationship between intestinal microbiota and lung diseases using bibliometric methods. This study aims to employ bibliometric techniques to quantitatively analyze research on the intestinal microbiota and the lung, providing a scientific basis for research directions and significant issues in this field.

## 2 Materials and methods

### 2.1 Data source and retrieval

We systematically searched the WoSCC database, setting the search timeframe from the database’s inception to 2023, with the search completed on October 25, 2023. A total of 1364 articles were retrieved, and the selection process was finalized on October 27, 2023. The search strategy was as follows:

1: [TI=(“gut microbiota” OR “intestinal microbiota” OR “fecal microbiota” OR “gastrointestinal microbiota” OR “gut microbiome” OR “intestinal microbiome” OR “fecal microbiome” OR “gastrointestinal microbiome” OR “intestinal bacteria” OR “gut bacteria” OR “fecal bacteria” OR “gastrointestinal bacteria” OR “intestinal flora” OR “gut flora” OR “fecal flora” OR “gastrointestinal flora” OR “gut microflora” OR “intestinal microflora” OR “fecal microflora” OR “gastrointestinal microflora”)] OR AB=(“gut microbiota” OR “intestinal microbiota” OR “fecal microbiota” OR “gastrointestinal microbiota” OR “gut microbiome” OR “intestinal microbiome” OR “fecal microbiome” OR “gastrointestinal microbiome” OR “intestinal bacteria” OR “gut bacteria” OR “fecal bacteria” OR “gastrointestinal bacteria” OR “intestinal flora” OR “gut flora” OR “fecal flora” OR “gastrointestinal flora” OR “gut microflora” OR “intestinal microflora” OR “fecal microflora” OR “gastrointestinal microflora”)
2: [TI=(“pulmonary” OR “lung”)] OR AB=(“pulmonary” OR “lung”)
3: #1 AND #2

**Figure 1:**
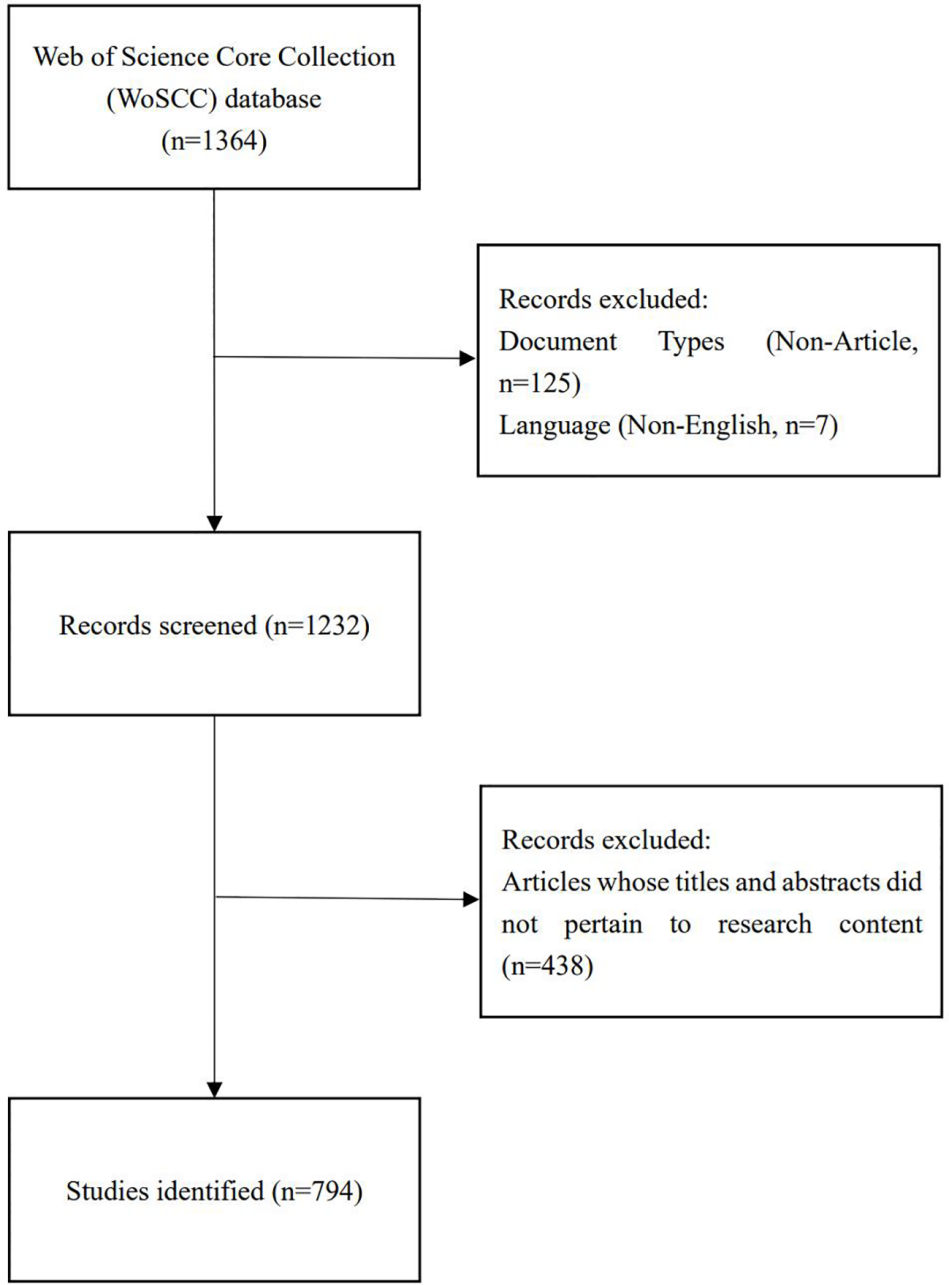
Flowchart of literature search selection.

Two reviewers independently identified and discussed potential discrepancies in the data search, ultimately reaching a consensus. After excluding articles written in non-English and those whose titles and abstracts did not match the research content, and by restricting the publication type to reviews and original articles, we obtained 794 articles that met the criteria for inclusion in the analysis (**Figure 1**).

This approach ensured a comprehensive and focused collection of relevant literature, providing a robust foundation for the subsequent bibliometric analysis.

### 2.2 Data collection

The original data were extracted from WoSCC database, including paper and citation numbers, publication year, country/region, affiliation, authors, journals, references, and keywords. The data were imported into VOSviewer (version 1.6.19) and CiteSpace (version 6.2.R3) for subsequent bibliometric analysis.

The Web of Science (WoS) Core Collection database, known for its rigorous journal selection, has become internationally recognized for evaluating the scientific achievements of scholars and institutions and for assessing the development of disciplines (Sun et al., 2022). Therefore, our research chose the WoS database.

CiteSpace is a software tool for visualizing and analyzing academic literature citation networks. It automatically generates citation network graphs and calculates citation frequency, keyword distribution, collaboration among authors, and displays this information in visual charts. CiteSpace also offers a range of analysis tools, such as clustering analysis and keyword co-occurrence analysis, to help researchers identify important research themes, hotspots, and influential papers in academic fields (Synnestvedt et al., 2005).

Similar to CiteSpace, VOSviewer supports visualization analysis based on literature citation networks. Using VOSviewer, researchers can easily explore citation relationships between literature and research hotspots. It aids in quickly grasping an overview of large-scale literature data, assisting in identifying field hotspots, research trends, and relevant literature resources and research directions related to specific research questions (van Eck and Waltman, 2010; Pan et al., 2018). In the generated network graphs, nodes can represent different countries, institutions, authors, or keywords. The size of a node reflects the number of publications associated with it, while different colors represent different clusters or years. Lines between nodes reveal collaboration or citation relationships.

Python is an open-source programming language known for its flexibility and productivity. Compared to traditional data visualization tools, Python offers the availability of multiple plotting and graphics libraries and the customization of various types of advanced charts (Wang and Maniruzzaman, 2022). Our study utilized Python to extract multiple key attributes from the literature, such as publication year, authors, institutions, countries, research fields, journals, references, and author keywords, followed by comprehensive statistical analysis. To visually represent the data, we also created bubble charts illustrating the annual publication trends in various research fields, journals, and author keywords. In these charts, the diameter of a bubble symbolizes the most prominent research field, journal, or author keyword in a particular year, and the number inside the bubble indicates the corresponding number of publications (Sun et al., 2022).

### 2.3 Data cleaning

Before initiating the bibliometric analysis, we meticulously processed the raw data using Python. Our data cleaning process involved several key steps:

- Country Consolidation: We merged countries that are geographically or politically part of the same entity. For example, Scotland, England, and Northern Ireland were combined under “United Kingdom”.
- Synonym Merging for Countries: We consolidated synonyms for countries, such as replacing “Peoples R China” and “Taiwan” with “China”.
- Standardization of Country Abbreviations: We standardized country abbreviations, for instance, converting “USA” to “United States”.
- Keyword Standardization: To avoid loss of information due to synonyms among keywords, we deduced author keywords and replaced synonyms with standardized terms.
- Author Identity Verification: We conducted thorough identity verification to eliminate confusion caused by authors with the same name. Besides using ORCID information for identity verification, we also referred to reliable sources such as official institutional websites.

These steps ensured the accuracy and consistency of our data, which is crucial for the integrity and reliability of our bibliometric analysis.

## 3 Results

### 3.1 The annual trend of paper publication quantity

**Figure 2:**
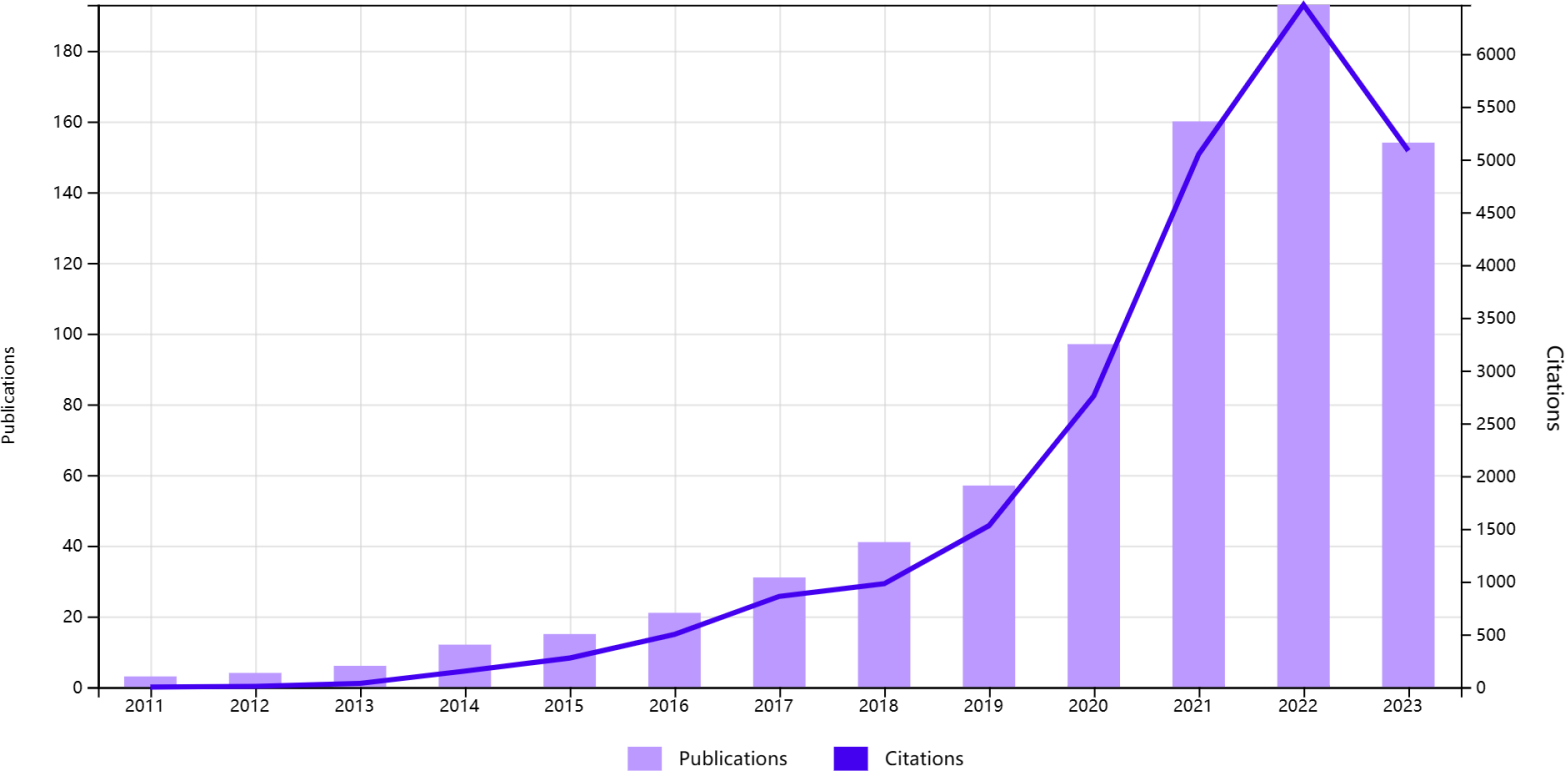
Trends in the growth of publications and the number of citations in intestinal microbiota and lung disease.

**Figure 2** illustrates the trends in annual publication volume and citation frequency. The total number of articles surged from 3 in 2011 to 190 in 2022. Since 2011, lung and intestinal microbiota research has shown an upward trend. The annual publication volume exceeded 150 articles starting in 2021, indicating a continuous increase in the number of scholars conducting related research and a flourishing development of theories in lung and intestinal microbiota research.

### 3.2 Analysis of authors

Since the database’s inception, 5,710 authors have participated in lung and intestinal microbiota research, publishing a total of 794 articles. **Table 1** displays the top 10 core authors, their publication count, total citations, and H-index. The top 10 authors published 63 articles collectively, totalling 1,750 citations. In terms of publication count, Jian Wang leads with 8 publications, followed by Derrick R Samuelson with 7 publications. Among them, 4,963 authors have published only one article, and only 23 have published five or more. Regarding total citations, Benjamin J Marsland leads with 2,522 citations, though outside the top 10 authors. Eva S Gollwitzer followed them with 2,120 citations and Aurelien Trompette with 2,052 citations. Regarding H-index, Valentin Sencio and Francois Trottein are in the leading positions. When assessing prolific authors, it is essential to consider the quantity of articles and the quality and publication timeline.

**Table 1:** Contribution of the top 10 authors in intestinal microbiota and lung diseases.

Rank	Author	Institution	Country	Publications	Citations	H-index
1	Wang, Jian	Univ Sci & Technol China	China	8	350	3
2	Samuelson, Derrick R.	Louisiana State Univ	United States	7	319	5
3	Sencio, Valentin	Univ Lille	France	6	398	6
3	Trottein, Francois	Univ Lille	France	6	398	6
3	Li, Jing	Univ FloridaUnited States		6	134	3
3	Zhang, Jun	Univ Iowa Hosp & Clin	United States	6	41	3
3	Li, Yalan	Univ Iowa Hosp & Clin	China	6	33	4
3	Peng, Guiying	Univ Iowa Hosp & Clin	China	6	33	4
3	Li, Na	Univ Iowa Hosp & Clin	China	6	29	3
3	Liu, Yang	Yunnan Univ Tradit Chinese Med	China	6	15	2

**Figure 3:**
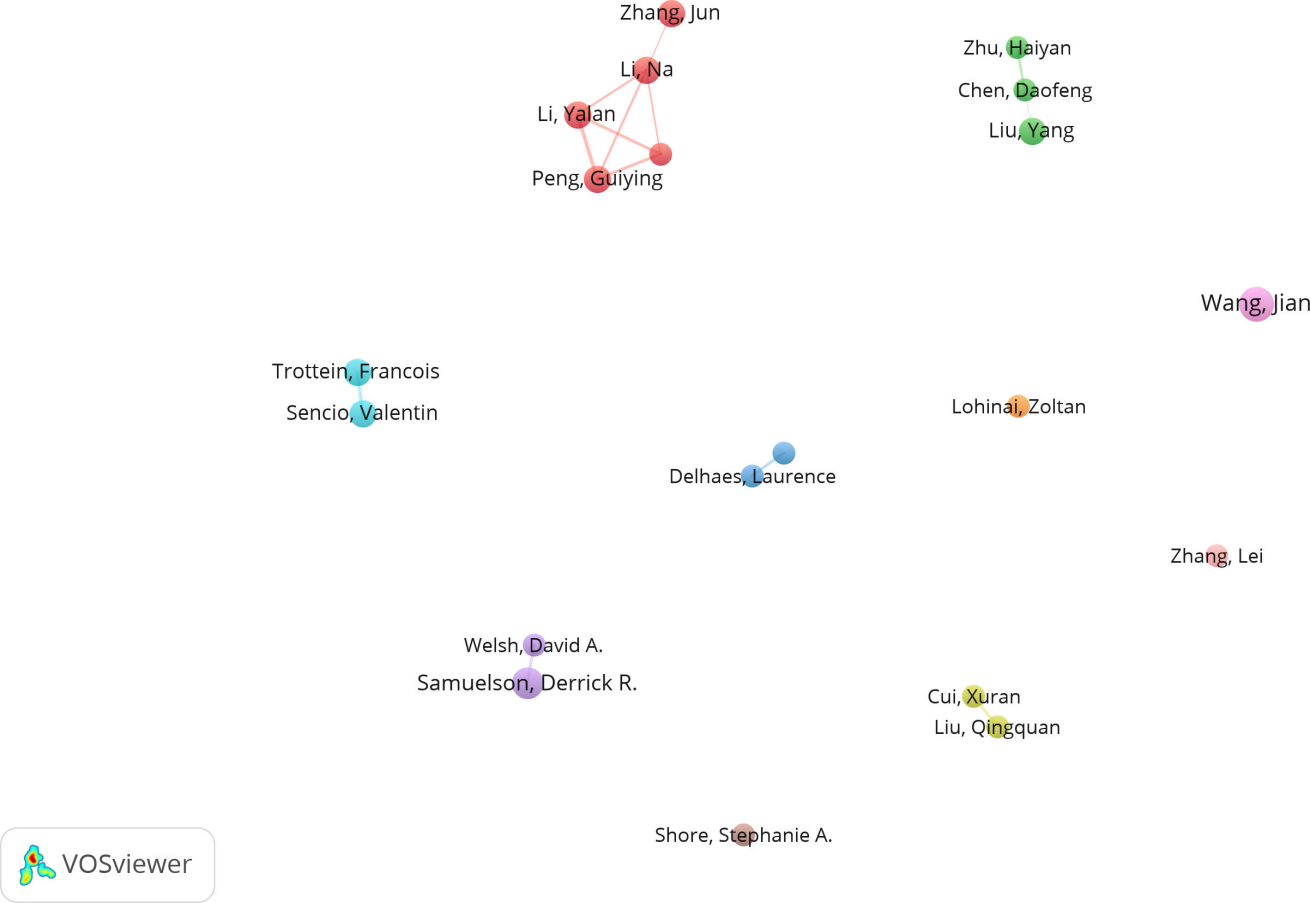
Cooperation map of top 20 authors in intestinal microbiota and lung diseases.

**Figure 4:**
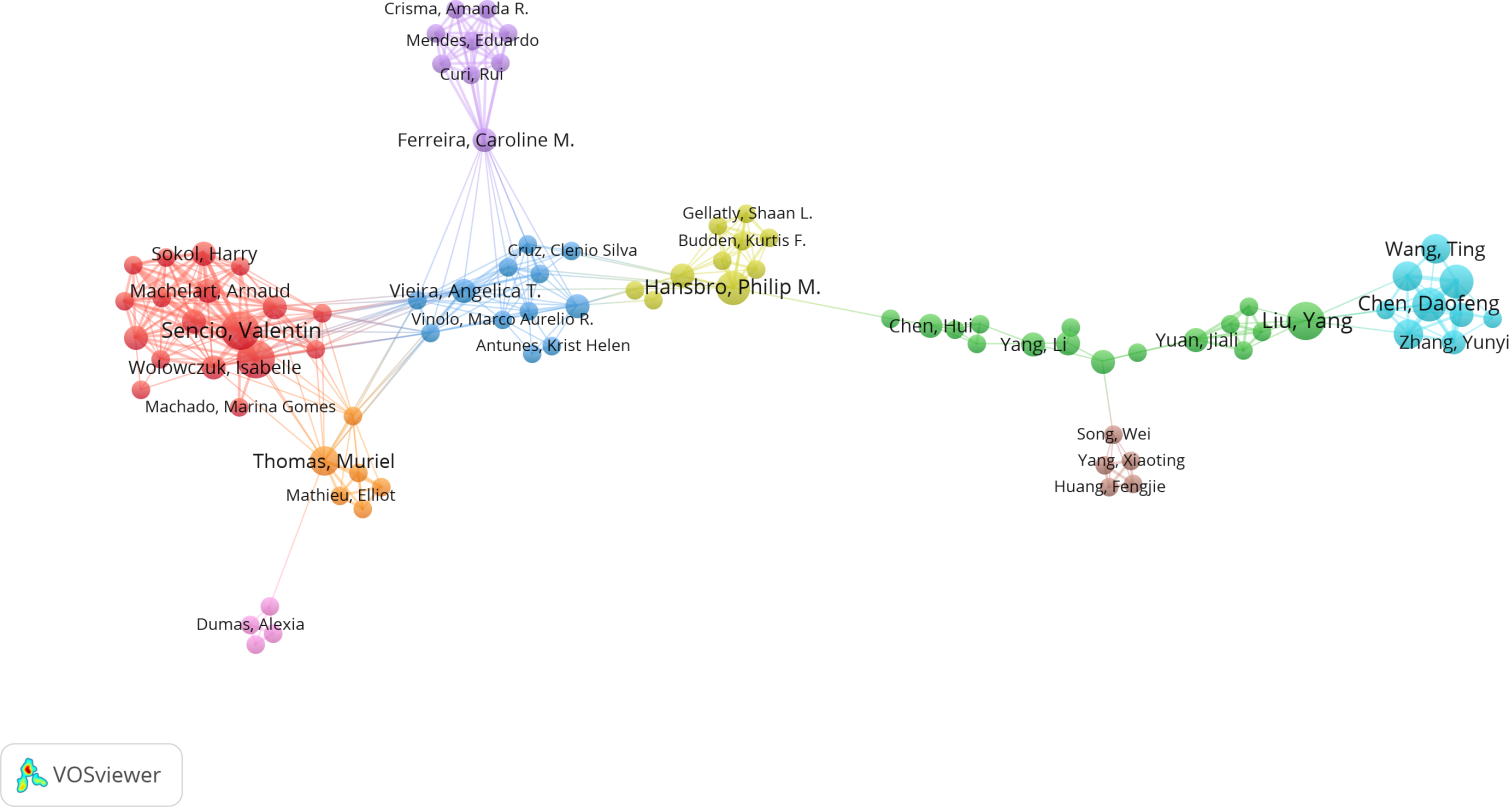
Cooperation map of authors in intestinal microbiota and lung diseases.

Among the top 10 authors in terms of publication volume, 5 are based in China, 3 in the United States, and 2 in France. This data indicates the concentration of research in this field in specific countries. VOSviewer software was used to depict the collaborations among authors in this field (**Figures 3**, **4**). We set a threshold in the statistical analysis, requiring authors to have published at least 2 articles; ultimately, only 720 authors met this threshold. After removing unconnected nodes, 89 authors met the threshold. In the network visualization, the size of a node increases with the number of contributions by the author. The larger the node, the more articles the author has published.

### 3.3 Analysis of institutions

1,431 institutions systematically published articles on the lung and intestinal microbiota. The majority of the top 10 institutions in terms of publication volume are from China. Zhejiang University in China ranks first in the number of publications and the H-index, with 26 publications, 878 total citations, and an H-index of 14. Fudan University is second with 18 publications, 297 total citations, and an H-index 8. Shanghai Jiao Tong University (17 publications, 470 total citations) and Sun Yat-sen University (17 publications, 310 total citations) are tied for third place (**Table 2**).

**Table 2:** Contribution of the top 10 institutions in intestinal microbiota and lung diseases.

Rank	Institution	Publications	Citations	H-index	Country
1	Zhejiang Univ	26	878	14	China
2	Fudan Univ	18	297	8	China
3	Shanghai Jiao Tong Univ	17	470	8	China
3	Sun Yat Sen Univ	17	310	7	China
4	Capital Med Univ	16	282	7	China
5	Beijing Univ Chinese Med	14	78	5	China
6	Univ Paris Saclay	13	804	9	France
6	Chinese Acad Sci	13	354	5	China
6	Guangzhou Med Univ	13	278	5	China
6	Cent South Univ	13	169	7	China

**Figure 5:**
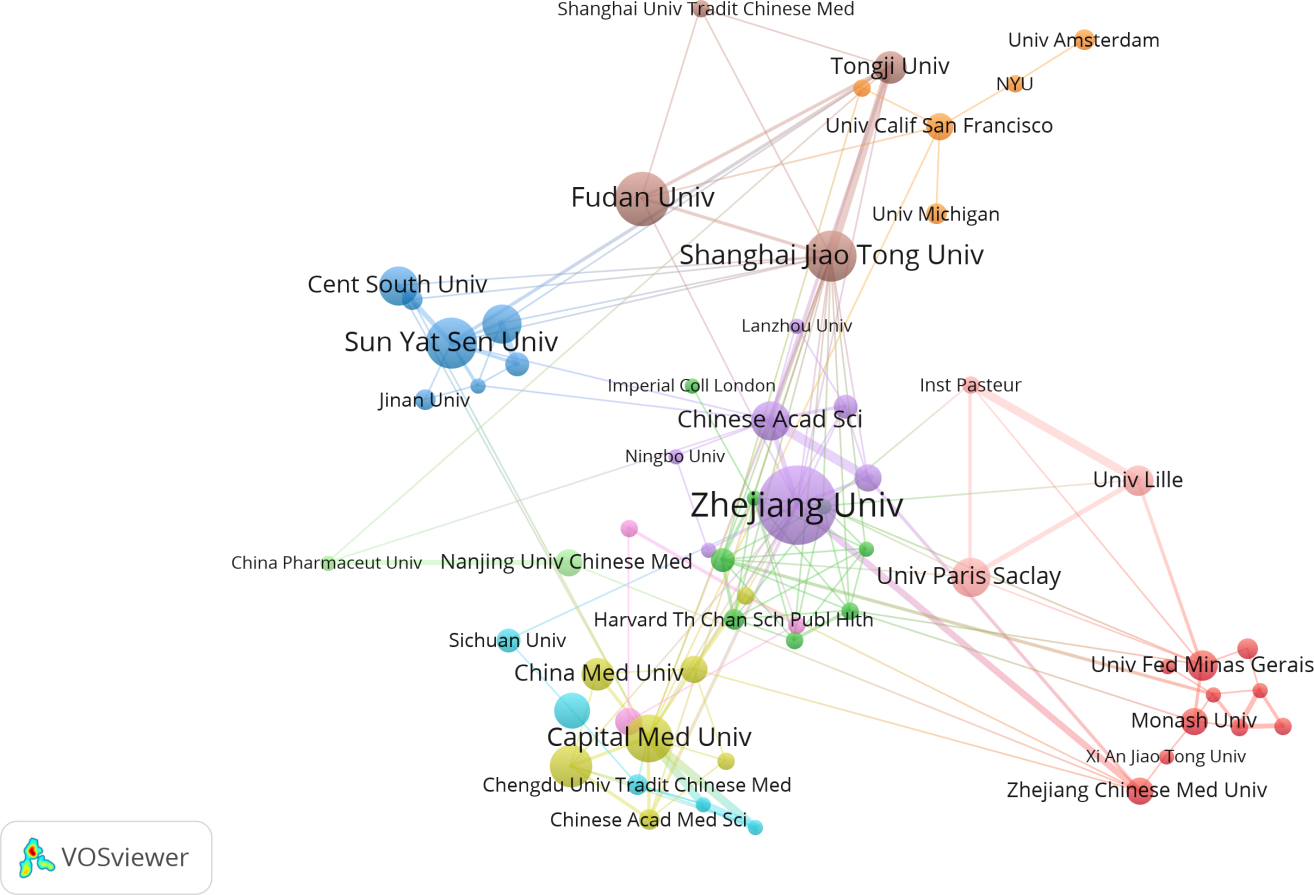
Cooperation map of institutions in intestinal microbiota and lung diseases.

Institutional collaboration analysis was conducted using VOSviewer software, and an institutional collaboration network map was constructed (**Figure 5**). The minimum publication threshold was set to 5, with 66 institutions meeting this threshold. After removing unconnected nodes, 61 institutions met the criteria. As shown in **Figure 5**, there is close collaboration among these institutions.

### 3.4 Analysis of countries/regions

**Figure 6:**
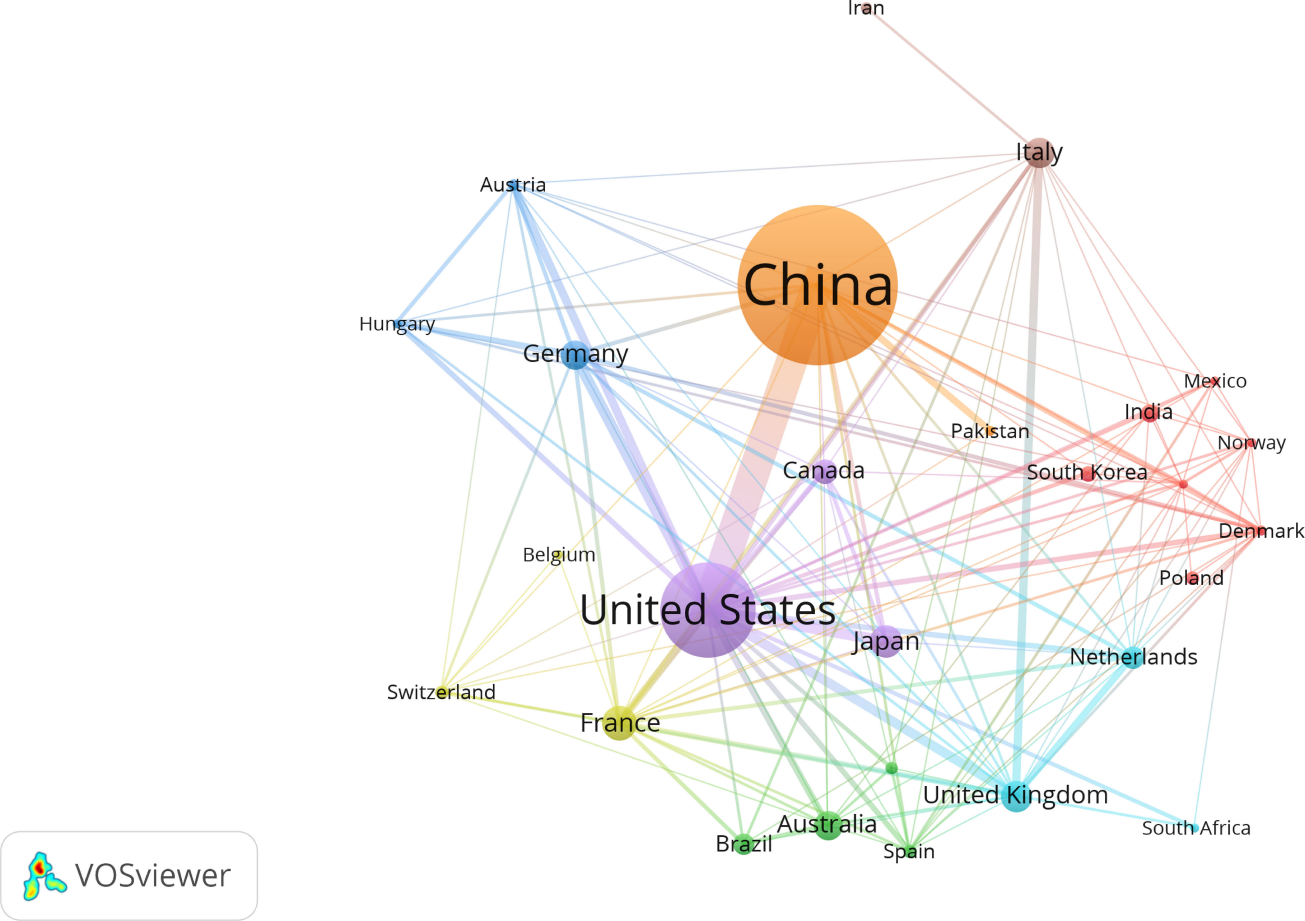
Cooperation map of countries/regions in intestinal microbiota and lung diseases.

Our analysis included 57 countries. The top 10 countries/regions were ranked based on the total number of publications by all authors. China had the highest publication volume, accounting for 45.2%, followed by the United States (21.8%) and France (5.3%) (**Table 3**, **Figure 6**). Only China and the United States published more than 100 articles. Although China had the highest number of publications, the United States led in total citations and H-index, indicating a higher quality of articles. The United States maintained the most vital international collaboration partnerships with other countries in this field.

**Table 3:** Contribution of the top 10 countries/regions in intestinal microbiota and lung diseases.

Rank	Country	Publications	Citations	H-index	Average Citations per Publication	Number of Cooperative Countries	Multinational Publications	Share of multinational cooperation publications
1	China	359	5411	36	15.07	21	40	11.14
2	United States	173	5604	41	32.39	33	77	44.51
3	France	42	2068	22	49.24	21	18	42.86
4	Japan	39	1188	17	30.46	6	15	38.46
5	United Kingdom	37	1408	18	38.05	23	25	67.57
6	Italy	35	923	15	26.37	18	17	48.57
7	Australia	33	2643	17	80.09	17	16	48.48
7	Germany	33	1278	15	38.73	14	18	54.55
8	Canada	27	1510	20	55.93	11	16	59.26
9	Netherlands	23	1019	13	44.3	15	18	78.26

### 3.5 Analysis of journals

794 articles related to the lung and intestinal microbiota were published across 333 journals, as detailed in **Table 4**. The journal with the most publications was *Frontiers in Microbiology* (39 articles, accounting for 4.9%), followed by *Frontiers in Immunology* (35 articles, 4.4%), *Frontiers in Cellular and Infection Microbiology* (23 articles, 2.9%), *Plos One* (21 articles, 2.6%), *Nutrients* (17 articles, 2.1%), *Biomedicine & Pharmacotherapy* (16 articles, 2.0%), *Scientific Reports* (15 articles, 1.9%), *International Journal of Molecular Sciences* (15 articles, 1.9%), *Gut Microbes* (13 articles, 1.6%), and *Frontiers in Pharmacology* (12 articles, 1.5%). *Nature Medicine* had the highest total citations (2004) but only published 3 articles.

**Table 4:** Contribution of the top 10 journals in intestinal microbiota and lung diseases.

Rank	Journal	Publications	Citations	Average Citations per Publication	The percentage of articles of institutions in total publications	IF
1	FRONTIERS IN MICROBIOLOGY	39	1168	29.95	4.01	5.2
2	FRONTIERS IN IMMUNOLOGY	35	943	26.94	3.6	7.3
3	FRONTIERS IN CELLULAR AND INFECTION MICROBIOLOGY	23	546	23.74	2.37	5.7
4	PLOS ONE	21	619	29.48	2.16	3.7
5	NUTRIENTS	17	201	11.82	1.75	5.9
6	BIOMEDICINE & PHARMACOTHERAPY	16	290	18.12	1.65	7.5
7	SCIENTIFIC REPORTS	15	425	28.33	1.54	4.6
7	INTERNATIONAL JOURNAL OF MOLECULAR SCIENCES	15	204	13.6	1.54	5.6
8	GUT MICROBES	13	542	41.69	1.34	12.2
9	FRONTIERS IN PHARMACOLOGY	12	58	4.83	1.23	5.6

**Supplementary Figure 1** presents a bubble chart of the top 20 journals related to lung and intestinal microbiota research. The chart reveals that since 2021, *Frontiers in Microbiology*, *Frontiers in Immunology*, and *Frontiers in Cellular and Infection Microbiology* have been the most prolific journals in this research area. *Plos One* has maintained a steady publication volume since 2013, while *Scientific Reports* shows a general downward trend in publication volume. Specifically, *Frontiers in Microbiology* published 12 articles in this field in 2023.

### 3.6 Analysis of research areas

The research on lung diseases and intestinal microbiota encompasses 64 fields, with **Table 5** listing the top 20 fields in publication volume. The fields of “Microbiology” and “Immunology” lead in publication volume, with 166 and 144 papers, respectively, and rank highest in total citations, with 6483 and 5025 citations each. The field with the highest average citation number is “Virology” (109.5 citations per paper), which, despite having a smaller publication volume of only 10 papers, has garnered relatively high attention and citations.

**Table 5:** Contribution of the top 10 research areas in intestinal microbiota and lung diseases.

Rank	Research Field	Publications	Citations	H-index	Average Citations per Publication	The percentage of articles of institutions in total publications
1	Microbiology	166	6483	41	39.05	17.08
2	Immunology	144	5025	37	34.9	14.81
3	Pharmacology & Pharmacy	89	944	16	10.61	9.16
4	Oncology	68	1544	20	22.71	7
5	Biochemistry & Molecular Biology	62	2999	19	48.37	6.38
6	Respiratory System	60	1395	19	23.25	6.17
7	Medicine, Research & Experimental	54	3226	20	59.74	5.56
8	Multidisciplinary Sciences	53	2140	20	40.38	5.45
9	Cell Biology	50	3257	21	65.14	5.14
10	Nutrition & Dietetics	42	553	13	13.17	4.32
11	Gastroenterology & Hepatology	29	1468	17	51.24	2.98
12	Medicine, General & Internal	27	324	11	12	2.78
13	Integrative & Complementary Medicine	25	116	7	4.64	2.57
14	Infectious Diseases	23	611	12	26.57	2.37
15	Food Science & Technology	21	240	8	11.43	2.16
16	Chemistry, Multidisciplinary	21	303	10	14.43	2.16
17	Biotechnology & Applied Microbiology	21	333	12	15.86	2.16
18	Environmental Sciences	16	110	6	6.88	1.65
19	Pediatrics	16	217	9	13.56	1.65
20	Toxicology	15	67	5	4.47	1.54

**Supplementary Figure 2** presents a bubble chart sorted by publication volume for the top 20 research fields. According to the chart, “Microbiology” and “Immunology” have been the most popular research fields since 2011. Since 2018, there has been a rising trend in the publication volume in fields such as “Pharmacology & Pharmacy,” “Oncology,” “Biochemistry & Molecular Biology,” “Respiratory System,” “Medicine, Research & Experimental,” “Multidisciplinary Sciences,” and “Cell Biology.” This trend indicates increasing attention in these areas.

### 3.7 Analysis of author keywords

An analysis was conducted on 1,046 author keywords, with “intestinal microbiota/microbiome,” “microbiota/microbiome,” and “gut-lung axis” being the keywords that appeared over 100 times (**Table 6**). As shown in the bubble chart of **Supplementary Figure 3**, since the outbreak of COVID-19/SARS-CoV-2 in 2020, it has become a focal point of interest.

**Table 6:** Contribution of the top 10 author keywords in intestinal microbiota and lung diseases.

Rank	Authorkeywords	Total Publications
1	intestinal microbiota/microbiome	333
2	microbiota/microbiome	178
3	gut-lung axis	132
4	COVID-19/SARS-CoV-2	81
5	probiotics	64
6	inflammation	60
7	asthma	57
8	short-chain fatty acids	47
9	lung cancer	44
10	dysbiosis	39

**Figure 7:**
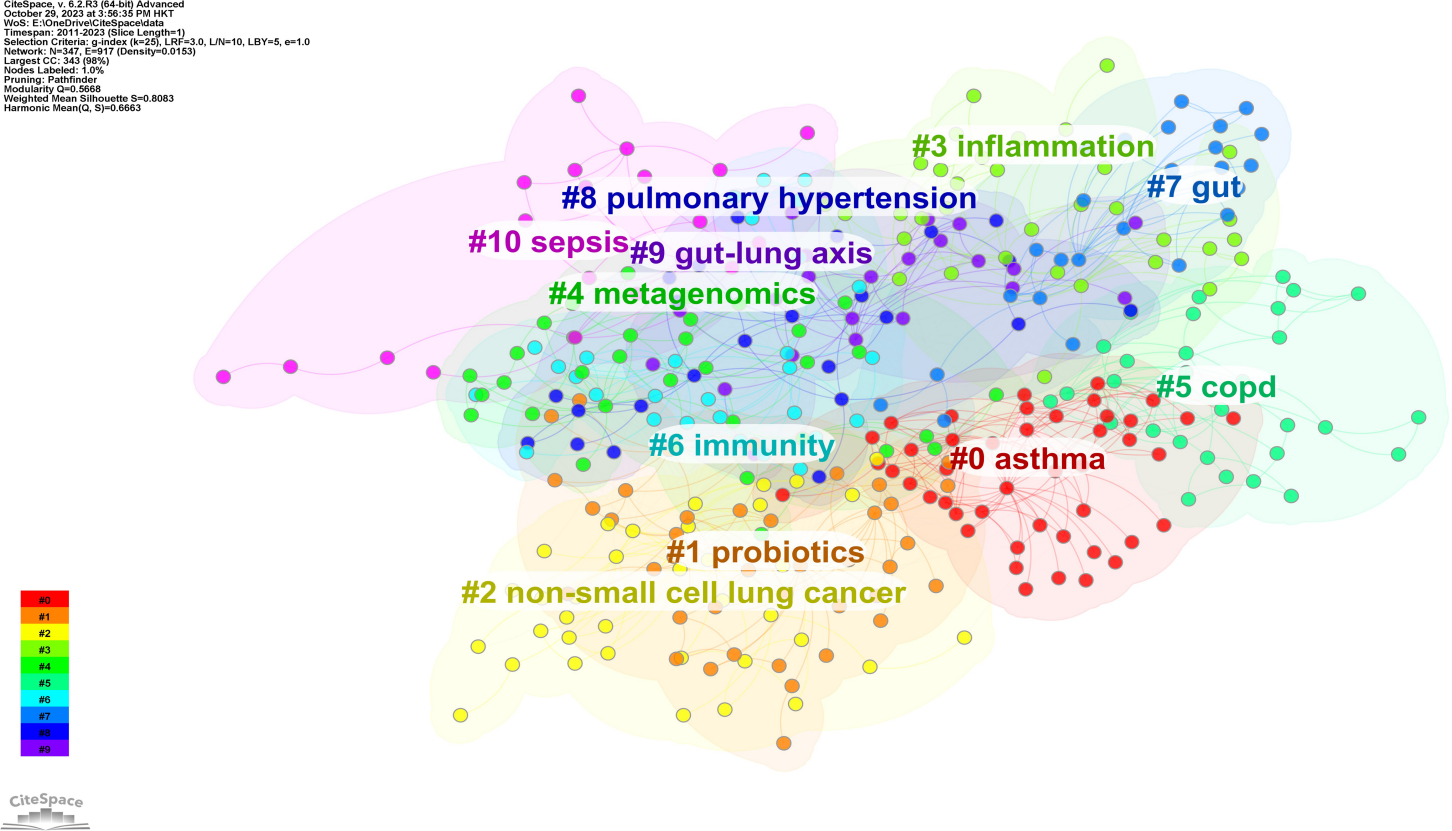
The clustered network map of author keywords in intestinal microbiota and lung diseases.

Using CiteSpace software, the keywords were categorized into 11 clusters (**Figure 7**). Closely related keywords were automatically grouped into a cluster, with each cluster named after the keyword with the highest log-likelihood ratio (LLR). The higher the LLR, the more representative the keyword is of its cluster. The 11 clusters identified in this field are #0 asthma, #1 probiotics, #2 non-small cell lung cancer, #3 inflammation, #4 metagenomics, #5 COPD, #6 immunity, #7 gut, #8 pulmonary hypertension, #9 gut-lung axis, and #10 sepsis, with cluster #0 being the largest.

**Figure 8:**
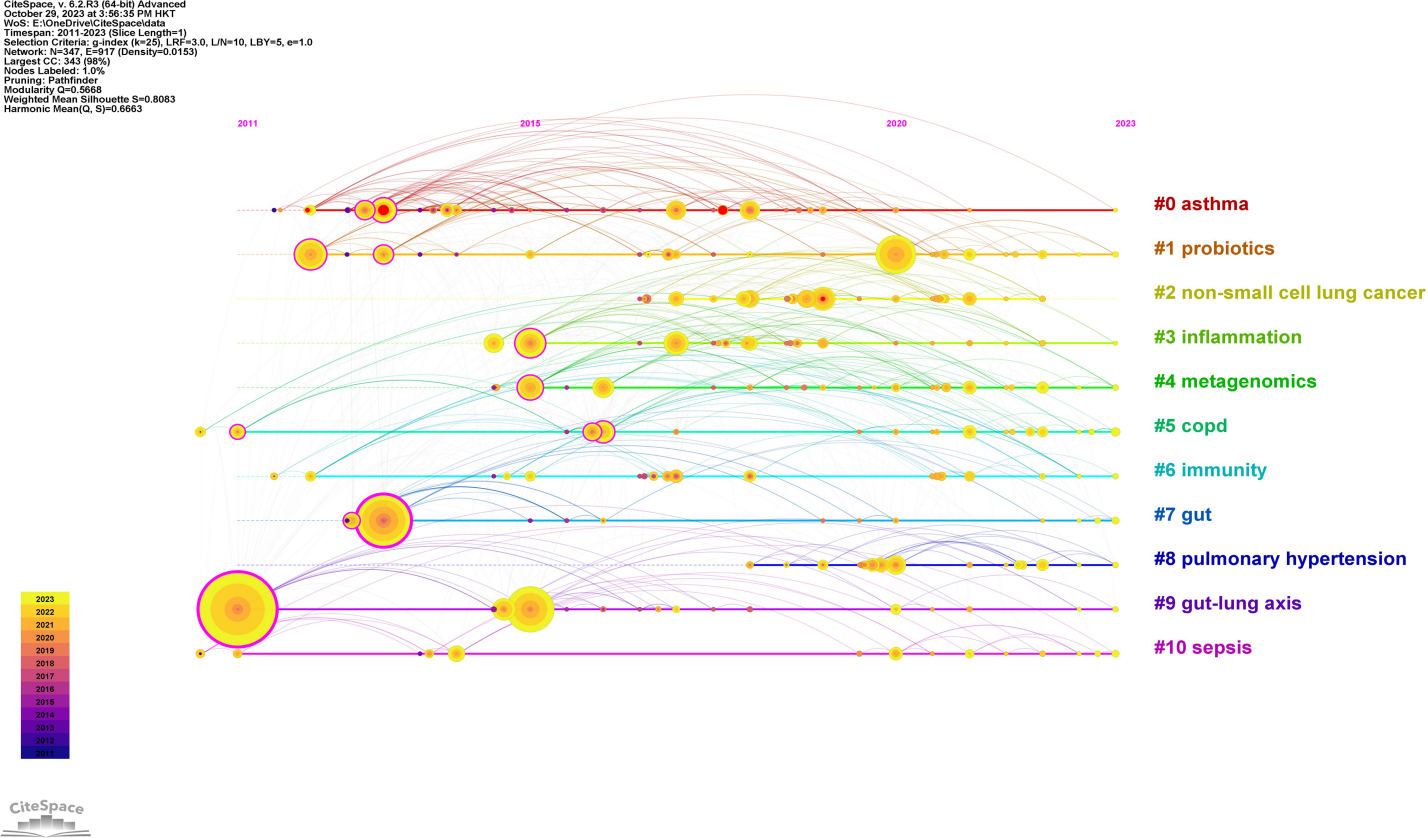
The timeline of clustered network map of author keywords in intestinal microbiota and lung diseases.

**Figure 9:**
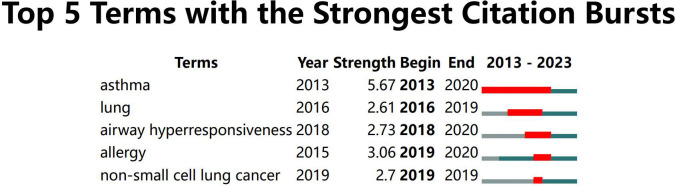
All author keywords with the strongest citation bursts in intestinal microbiota and lung diseases.

A timeline chart was created based on the first year of the appearance of the keywords (**Figure 8**). In this field, the earliest active cluster was #9, followed by the rise of #1. Our study identified 5 keywords with the most potent citation bursts (**Figure 9**), including asthma, lung, airway hyperresponsiveness, allergy, and non-small cell lung cancer.

### 3.8 Analysis of ESI highly cited papers and citation bursts references

**Figure 10:**
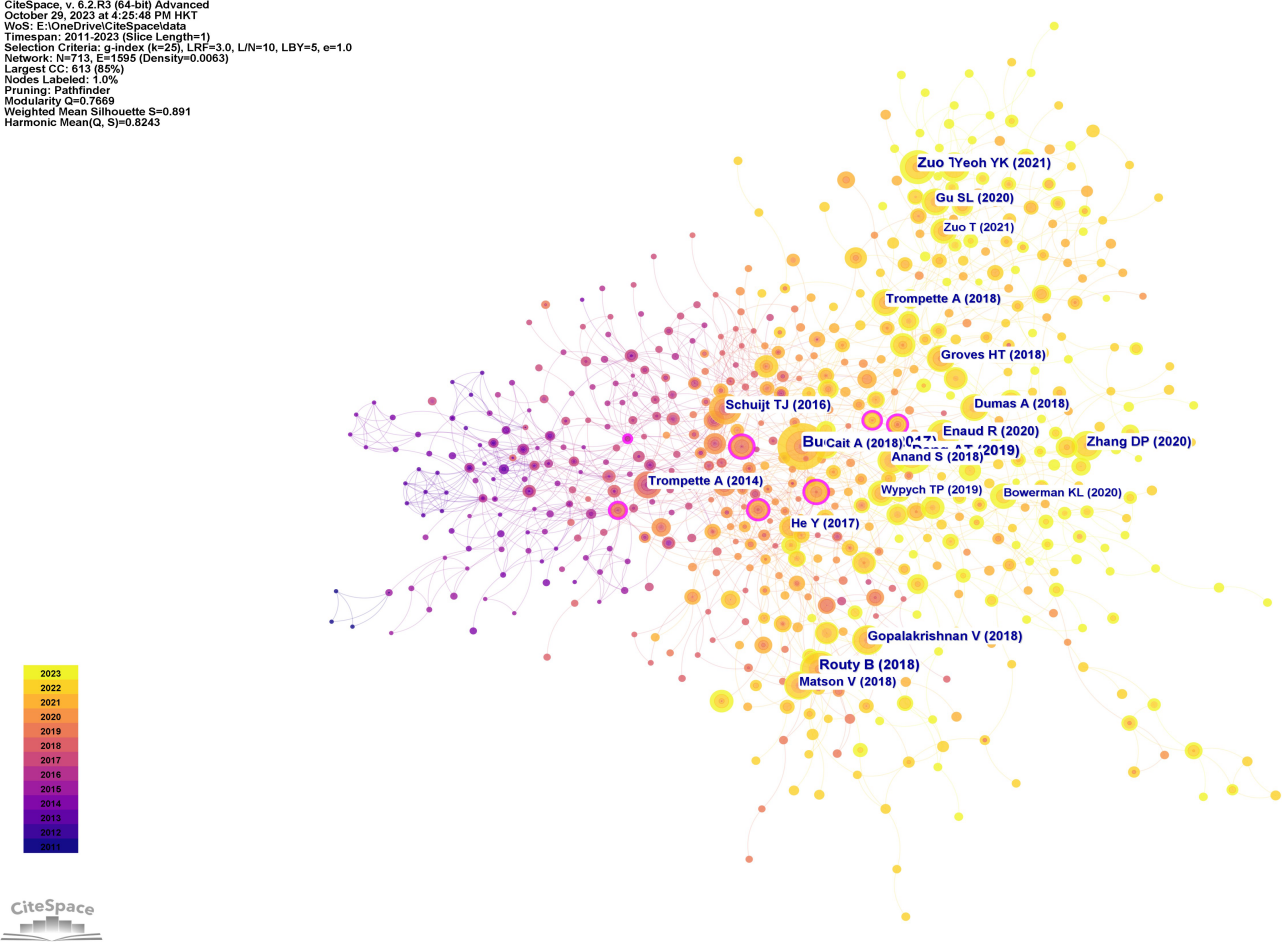
Reference co-citation network in intestinal microbiota and lung diseases.

**Figure 11:**
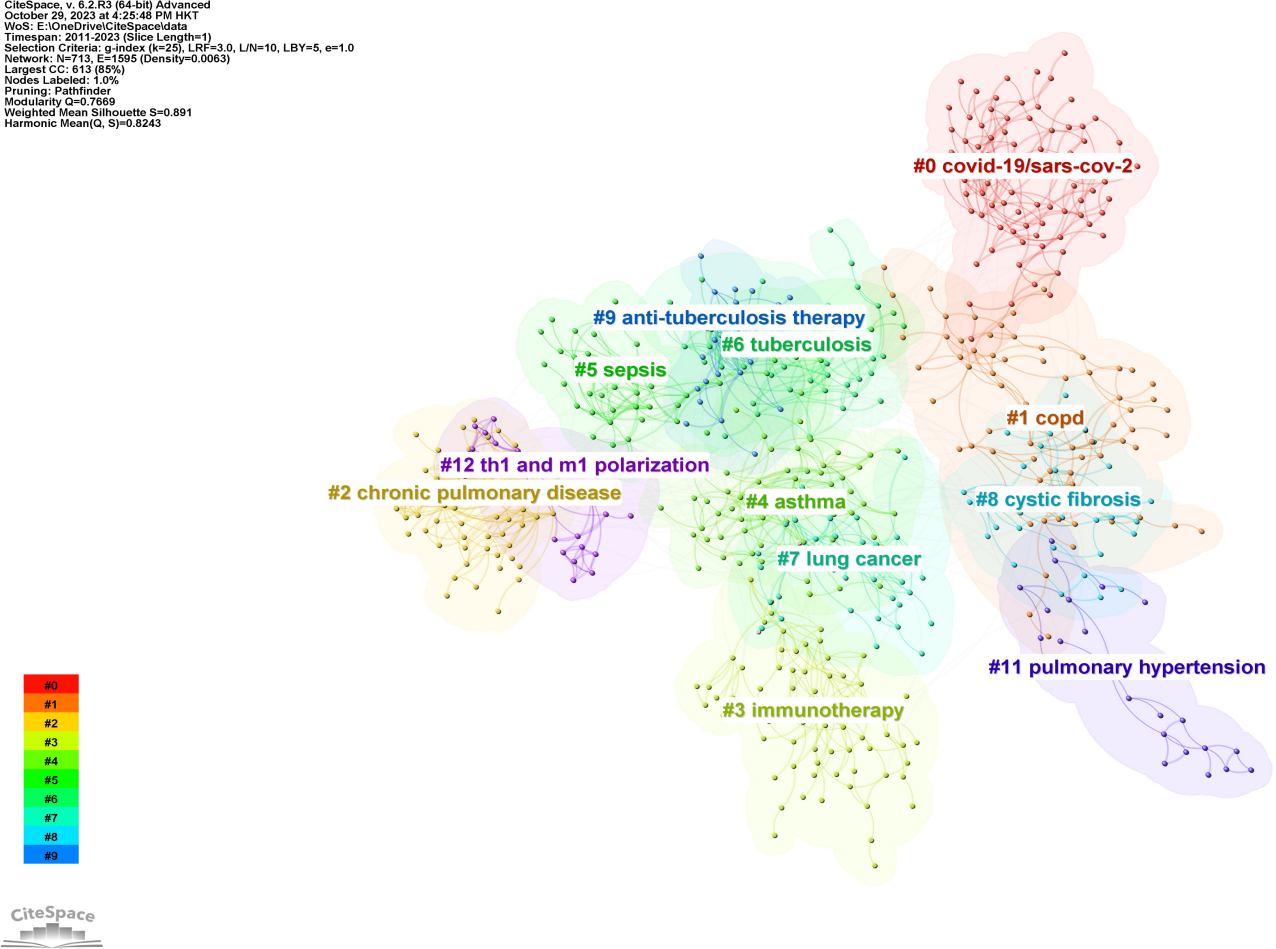
The clustered network map of co-cited references in intestinal microbiota and lung diseases.

**Figure 10** displays a network visualization map of cited references, with yellow nodes representing the most recently appearing keywords, indicating these are the latest cited references in the field. **Figure 11** shows 12 related clusters identified by CiteSpace: COVID-19/SARS-CoV-2, COPD, chronic pulmonary disease, immunotherapy, asthma, sepsis, tuberculosis, lung cancer, cystic fibrosis, anti-tuberculosis therapy, pulmonary hypertension, and Th1 and M1 polarization.

**Figure 12:**
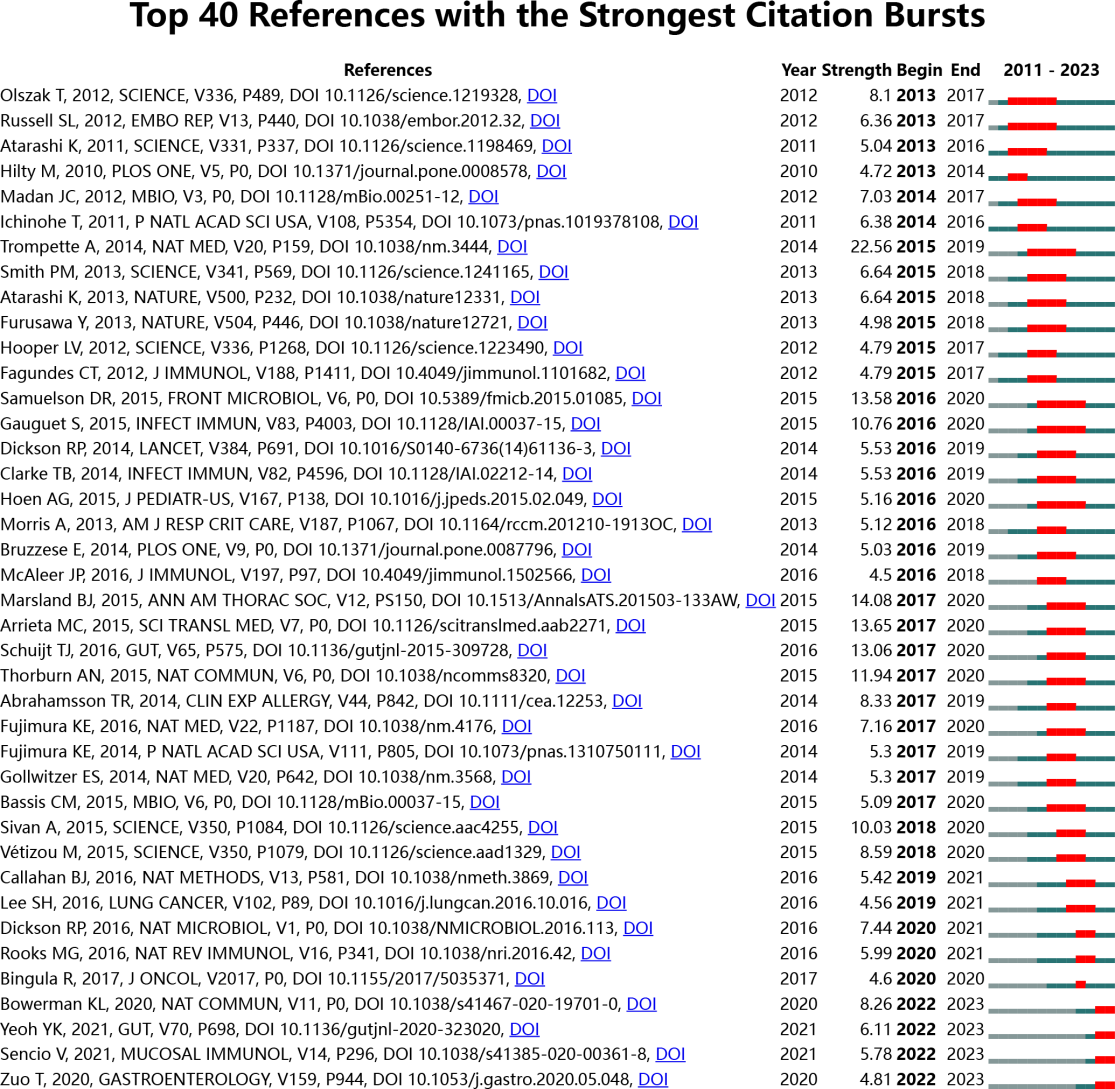
Top 40 references with the strongest citation bursts in intestinal microbiota and lung diseases.

A burst strength analysis of the references was conducted using CiteSpace to gain a comprehensive understanding of the cited references. The time frame was set from 2011 to 2023, with a one-year interval, and 40 references with significant burst strength were identified. Among these, 8 references had a burst strength more significant than 10 (**Figure 12**). This analysis highlights the most influential and rapidly emerging topics in the field within the specified time frame.

## 4 Discussion

### 4.1 General information

In this study, we analyzed 794 articles from the WoSCC database in the field of lung diseases and intestinal microbiota using Python, VOSviewer, and CiteSpace. The research findings indicate a significant increase in annual publications and citation numbers starting from 2020, with a predominant contribution from China. This highlights the extensive research contributions of China in this field. Among the top 10 institutions in terms of publications, 9 are from China, including Zhejiang University, Fudan University, Shanghai Jiao Tong University, Sun Yat-sen University, Capital Medical University, Beijing University of Traditional Chinese Medicine, Chinese Academy of Sciences, Guangzhou Medical University, and Southeast University. This indicates increasing attention and emphasis on this research area in China.

In terms of publication volume, the most prolific author is Jian Wang from China. However, in terms of total citations, Benjamin J Marsland from Monash University, Australia, holds the leading position but is outside the top 10 authors. Following him are Eva S Gollwitzer from Universitaire Vaudois, Switzerland, and Aurelien Trompette. In terms of the H-index, the leading positions are occupied by Valentin Sencio and Francois Trottein. When assessing prolific authors, it is crucial to consider the quantity of their publications and the quality of the articles.

From the perspective of journals, *Frontiers in Microbiology* holds a leading position as the journal with the highest number of publications in this field. However, *Nature Medicine* has the highest total citations despite publishing only three articles. This discrepancy highlights the significant impact and high quality of the articles in *Nature Medicine*.

The most focused research fields are “Microbiology” and “Immunology”, which lead in both the number of publications and total citations. Despite having fewer publications, the field of “Virology” has the highest average citation count. This heightened attention and citation rate could be related to the outbreak of COVID-19/SARS-CoV-2 in 2020, which has also become a focal point of interest.

Benjamin J Marsland from Monash University is a prominent author in terms of citations in this field. His long-term research into the impact of the microbiome on human immunity and the respiratory system has been influential. His team discovered a new protective pathway within the gut-lung axis - L-tyrosine–PCS, which can be used to treat or prevent inflammatory diseases (Wypych et al., 2021). This discovery underscores the critical interplay between intestinal microbiota and lung health, revealing novel pathways for therapeutic interventions.

### 4.2 Research hotspots and trends

The lung and intestinal microbiota fields have consistently attracted attention from scholars worldwide. Current research hotspots, identified through analyses of the most-cited references, keyword co-occurrence, clustering, and emerging trends, primarily focus on the gut-lung axis, probiotics, COVID-19, asthma, and COPD. It is well-recognized that intestinal microbiota dysbiosis is associated with increased susceptibility to respiratory diseases and alterations in lung immune responses and homeostasis. Although an increasing number of epidemiological and experimental studies have highlighted the relationship between the intestinal microbiome and the lungs, referred to as the “gut-lung axis” (Bingula et al., 2017; Dang and Marsland, 2019), the mechanisms by which intestinal microbiota influence lung diseases remain unclear (Budden et al., 2017). Understanding these mechanisms is crucial for developing effective treatments and prevention strategies for respiratory diseases influenced by intestinal microbiota.

#### 4.2.1 SCFAs

The human gut harbors a rich and diverse microbial community, predominantly constituted by the intestinal microbiota. The dominant phyla within this community include Firmicutes (e.g., Clostridia, Lactobacillus) and Bacteroidetes (e.g., Bacteroides, Prevotella) (Adak and Khan, 2019). Research into the human microbiome has shown that the intestinal microbiota plays a crucial role in maintaining homeostasis in the gastrointestinal system and distant organs by modulating immune responses. It is critical to host health and intestinal immune homeostasis (Tan et al., 2014). However, intestinal microbiota dysbiosis can impact lung diseases, such as pneumonia, asthma, lung cancer, COPD (Chronic Obstructive Pulmonary Disease), and PH (Pulmonary Hypertension). This influence is likely related to alterations in immune responses, hormonal balance, and metabolic homeostasis (Bander et al., 2020; Ma et al., 2022).

Short-chain fatty acids (SCFAs) are among the most abundant microbial metabolites in the gut, playing fundamental roles such as lowering intestinal pH and promoting mucin synthesis. These functions prevent the adherence of harmful bacteria, enhance epithelial integrity, and strengthen the host’s immune system (Ashique et al., 2022). SCFAs interact with G-protein-coupled receptors (GPCRs) and regulate the differentiation of Treg, Th1, and Th17 cells. They also inhibit histone deacetylases (HDACs), thereby controlling cytokine storms and excessive immune responses, making them essential regulators in immune responses, inflammation, and the development of lung diseases (Jardou and Lawson, 2021; Smith et al., 2013; Castro-Mejía et al., 2018; Kleiner et al., 2015; Tang et al., 2021; Brown et al., 2017). However, not all studies report the anti-inflammatory actions of SCFAs. They have also been shown to increase the production of pro-inflammatory cytokines in cells stimulated by toll-like receptors (TLRs) (Rutting et al., 2019; Mirmonsef et al., 2012). Further research by Ghorbani et al. (2015) suggests that SCFAs can have anti-inflammatory and pro-inflammatory effects on lung cells. This dual role indicates that the therapeutic value of SCFAs in treating and managing diseases, particularly those involving the lung, is a promising area for further exploration. The complex nature of SCFA interactions in the human body, especially in the context of the gut-lung axis, highlights the importance of a nuanced understanding of these metabolites in health and disease.

#### 4.2.2 Airway microbiome

Research suggests that gut microbiota influence the composition of airway microbiota through their metabolic products, such as short-chain fatty acids (SCFAs), and immune cells (Reyes et al., 2012; Chakradhar, 2017; Cait et al., 2018; Steed et al., 2017; Samuelson et al., 2015; Herbst et al., 2011; Grayson et al., 2018). Compared to gut microbiota, research on airway microbiota is still in its infancy, but interest in this area is gradually increasing (Dang and Marsland, 2019). Rapid advancements in high-throughput sequencing technologies have corrected the misconception that “healthy lungs are sterile,” making airway microbiomics a focus of current research. Multiple studies have confirmed that the lungs are not a sterile environment, although the microbial load in the airways is significantly lower than in the gut (Yagi et al., 2021; Rastogi et al., 2022). A mature airway microbiome is equally important for the development and regulation of adaptive and innate immune responses (Enaud et al., 2020; Pattaroni et al., 2018). Increasingly, research is showing a link between airway microbiota and respiratory diseases (such as ventilator-associated pneumonia, asthma, chronic obstructive pulmonary disease) as well as metabolic diseases (such as obesity, diabetes) (Shah and Bunyavanich, 2021; Huang and Shi, 2019; Tiew et al., 2021; Lee et al., 2019; Martin-Loeches et al., 2020; Trompette et al., 2014; Fenn et al., 2022). Comparing the airway microbiome composition of healthy individuals and patients with diseases can reveal specific microbial communities associated with the onset and progression of diseases. Reviews by Yagi et al. (2021) suggest that the airway microbiome could become a useful biomarker for respiratory diseases in clinical settings. Therefore, investigating the role of airway microbiota dysbiosis in various lung diseases is crucial.

Researchers have delved into the functions of airway microbiota within the body. They have found that certain microbes can positively impact respiratory health by producing specific metabolic products or participating in immune responses. Additionally, airway microbiota interact with host cells, influencing disease progression by regulating host gene expression (Yuan et al., 2022; Dang and Marsland, 2019; Ver Heul et al., 2019; Yan et al., 2022; Sequeira et al., 2020; Brown et al., 2017; Subramaniam et al., 2015). With the widespread use of broad-spectrum antibiotics, increasing numbers of antibiotic-resistant strains are emerging in the airway microbiota. Researchers are studying the transmission pathways of antibiotic resistance genes in airway microbiota and the mechanisms of resistant strain formation to better manage antibiotic use and develop new treatment strategies (Lin et al., 2023; Cai et al., 2022; Lin et al., 2021; Chmiel et al., 2014; Sherrard et al., 2014; Mac Aogáin et al., 2020). Recent research indicates that the airway microbiome could spread through airborne transmission, direct contact, or other routes among people, particularly since the COVID-19 outbreak (Doggett et al., 2020). This finding is significant for disease control, as intervening in the transmission pathways of the airway microbiome could help prevent the spread of diseases.

#### 4.2.3 Probiotics

Keyword cluster analysis has revealed that probiotics are an emerging research hotspot in the study of the relationship between intestinal bacteria and lung diseases. Probiotics, beneficial microorganisms, selectively stimulate the growth and activity of a limited number of bacteria in the gut (da Silva et al., 2021). Primarily, this refers to species within the Lactobacillus and Bifidobacterium genera in the intestine. Probiotics play multiple roles within the human body: they aid in breaking down complex substances in food, promoting digestion and absorption. Additionally, they produce enzymes and substances that enhance the utilization of nutrients in food. Probiotics can competitively occupy ecological niches in the gut, inhibiting the growth of harmful bacteria and reducing pathogenic attacks and infections on the host. They also stimulate and regulate the function of the host’s immune system, enhancing the body’s immunity and helping to fend off diseases (Reid et al., 2017; Licciardi et al., 2012). Probiotics produce beneficial metabolic products, such as short-chain fatty acids, which regulate the intestinal pH balance and maintain gut health (Kim et al., 2019; Ragan et al., 2022; Yan and Polk, 2020). Modulating the composition of the gut microbiota, adjusting the metabolic products of gut microbes, and improving intestinal barrier function are potential regulatory mechanisms of probiotics (Li et al., 2021; Wang X. et al., 2021). Professor Matsuzaki demonstrated the efficacy of probiotics as early as 1985 (Matsuzaki et al., 1985). However, current research on the role of probiotics in lung diseases is relatively limited, and more studies are needed to confirm their effects in this context.

### 4.3 Future research directions

The gut-lung axis is an emerging field with many aspects yet to be fully understood. Future research aims to delve deeper into understanding the gut microbiota and its metabolic products, airway microbiota and their metabolites, and to develop suitable bioinformatics and machine learning models. These models are intended to predict and assess various gut-lung axis diseases and health statuses, leading to the development of personalized prevention and treatment strategies (Zheng et al., 2020; Zheng et al., 2023; Liang et al., 2022). Recent discoveries indicate significant differences in the gut microbiota of lung cancer patients compared to their lung microbiome communities. Changes in the gut microbiota can affect the prognosis and treatment outcomes of lung cancer (Goto, 2022; Tsay et al., 2021; Tsay et al., 2018), making the role of the gut-lung axis in the prevention, diagnosis, and treatment of lung cancer a valuable area of exploration. Dietary habits have been found to influence the gut microbiota, thereby impacting the prevention and treatment of lung diseases (Gasmi et al., 2021; Healy et al., 2021). Understanding the relationship between nutrition and the gut-lung axis, and designing specific dietary plans, may be one of the future research directions. COVID-19, one of the most widely discussed diseases in recent years, has been shown to be influenced by the gut microbiota, which may affect the host’s infection and immune response to the virus (Wang H. et al., 2021; McGinniss and Collman, 2021; Liu et al., 2022; Ancona et al., 2023). Therefore, studying the relationship between the gut-lung axis and COVID-19 is of significant importance for the prevention and treatment of this disease. This line of research holds the potential to unlock new insights into managing not only COVID-19 but also other respiratory diseases influenced by the complex interactions within the gut-lung axis.

### 4.4 Strengths and limitations

Our study leveraged bibliometric methods to perform a visual analysis of research on the relationship between intestinal microbiota and lung diseases, aiding scholars in better understanding the hotspots and trends in this field. However, our study also has limitations. We analyzed only articles published on WoSCC within a specific timeframe and in English, potentially overlooking research results from other databases. Despite this, using visual methods to understand the current state, hotspots, and trends in a field remains valuable.

## 5 Conclusion

Using Python programming, VOSviewer, and CiteSpace software, we conducted a bibliometric study on articles related to the gut microbiota and lung diseases. The results indicate an upward trend in research volume in this field since 2011, signifying the burgeoning development of theories related to the lung and gut microbiota. China has the highest total publication volume in this area, while the quality of articles from the United States is comparatively higher. The correlation and potential mechanisms between the gut microbiota and lung diseases, including asthma, COPD, lung cancer, and respiratory infections, remain hot research topics. However, the understanding of the mechanisms involved in the gut-lung axis is still in its nascent stages and requires further elucidation. Future research needs to focus more on the gut microbiota and airway microbiota to enhance our understanding of the gut-lung axis. This deeper understanding is crucial for developing new and effective treatment strategies for lung diseases. As this field continues to evolve, it’s evident that interdisciplinary research encompassing microbiology, immunology, and respiratory medicine will be pivotal in unlocking new insights into the gut-lung axis and its implications for human health.

## Data availability statement

The original contributions presented in the study are included in the article/**Supplementary Material**. Further inquiries can be directed to the corresponding authors.

## Author contributions

WS: Writing – original draft. TZ: Writing – review & editing. PD: Writing – review & editing. LG: Writing – review & editing. XZ: Writing – review & editing. KL: Writing – review & editing.

## Supplementary material

Supplementary Figure 1 Bubble chart of the top 20 journals by year.

Supplementary Figure 2 Bubble chart of the top 20 research areas by year.

Supplementary Figure 3 Bubble chart of the top 30 author keywords by year.
