# Supplementary figures and images for: Bibliometric analysis of intestinal microbiota and lung diseases
Source: Front Cell Infect Microbiol. 2024 Feb 15;14:1347110. doi: 10.3389/fcimb.2024.1347110 (PMC10902173; doi:10.3389/fcimb.2024.1347110)

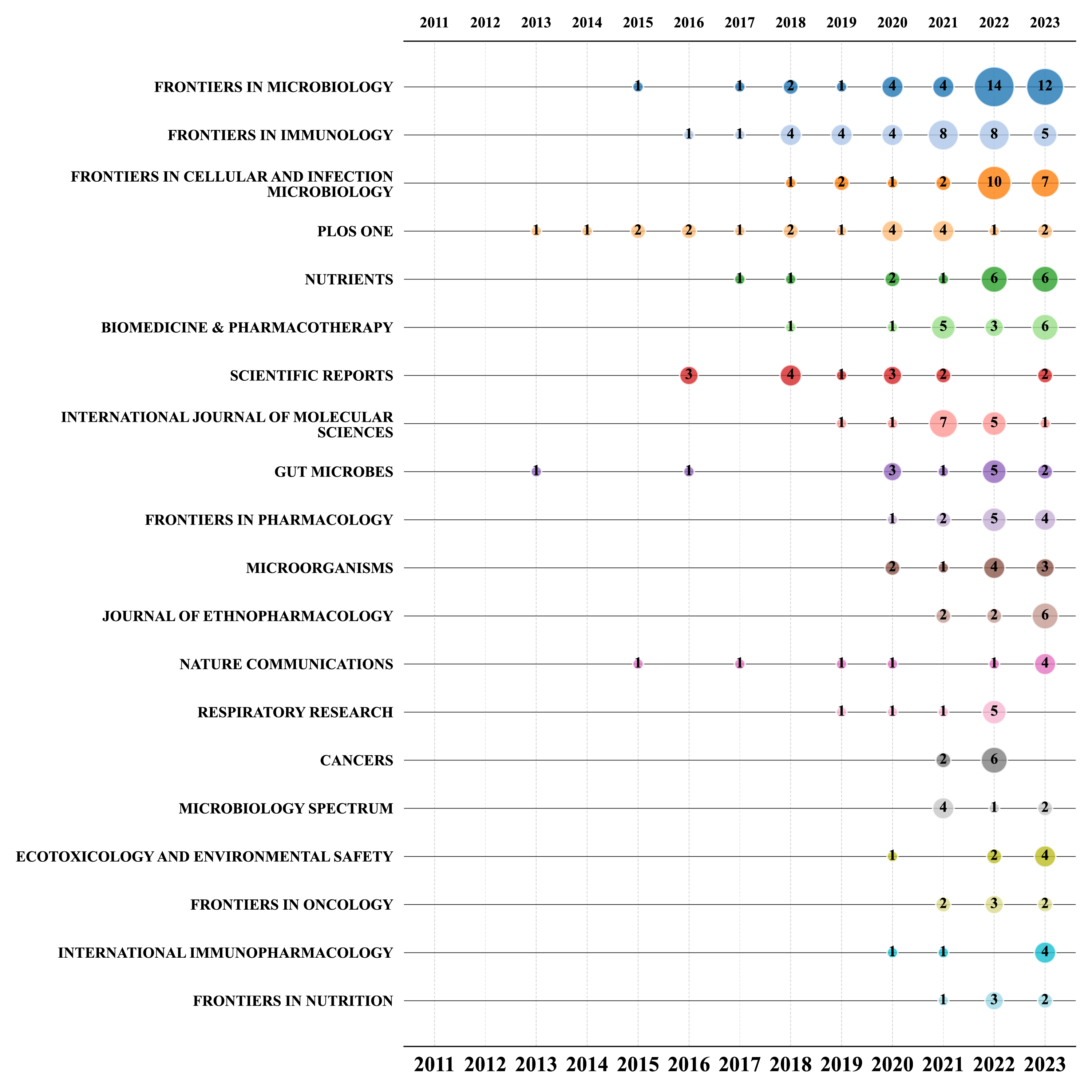

Supplement: Supplementary Figure 1 — Bubble chart of the top 20 journals by year. [file Image_1.tif]

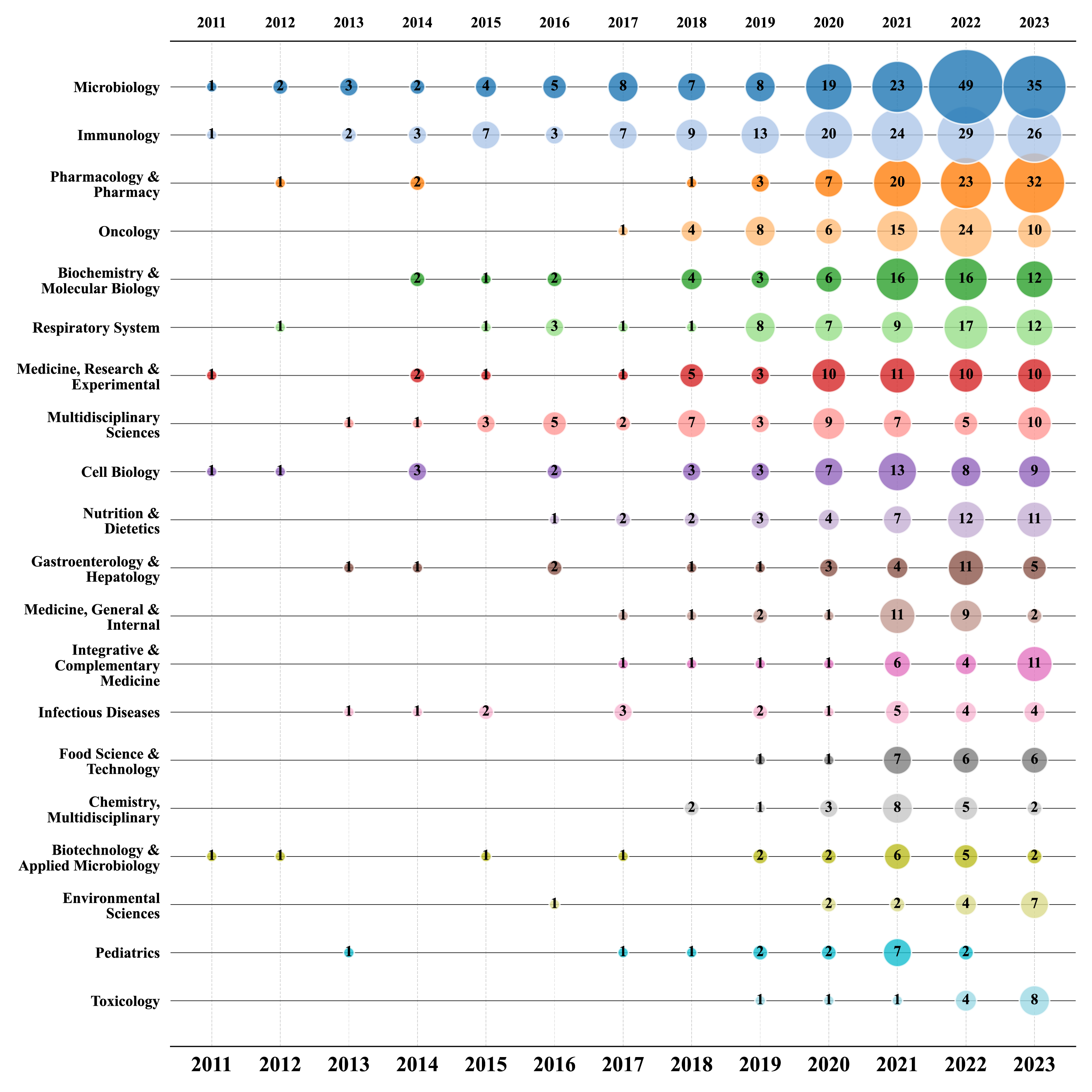

Supplement: Supplementary Figure 2 — Bubble chart of the top 20 research areas by year. [file Image_2.tif]

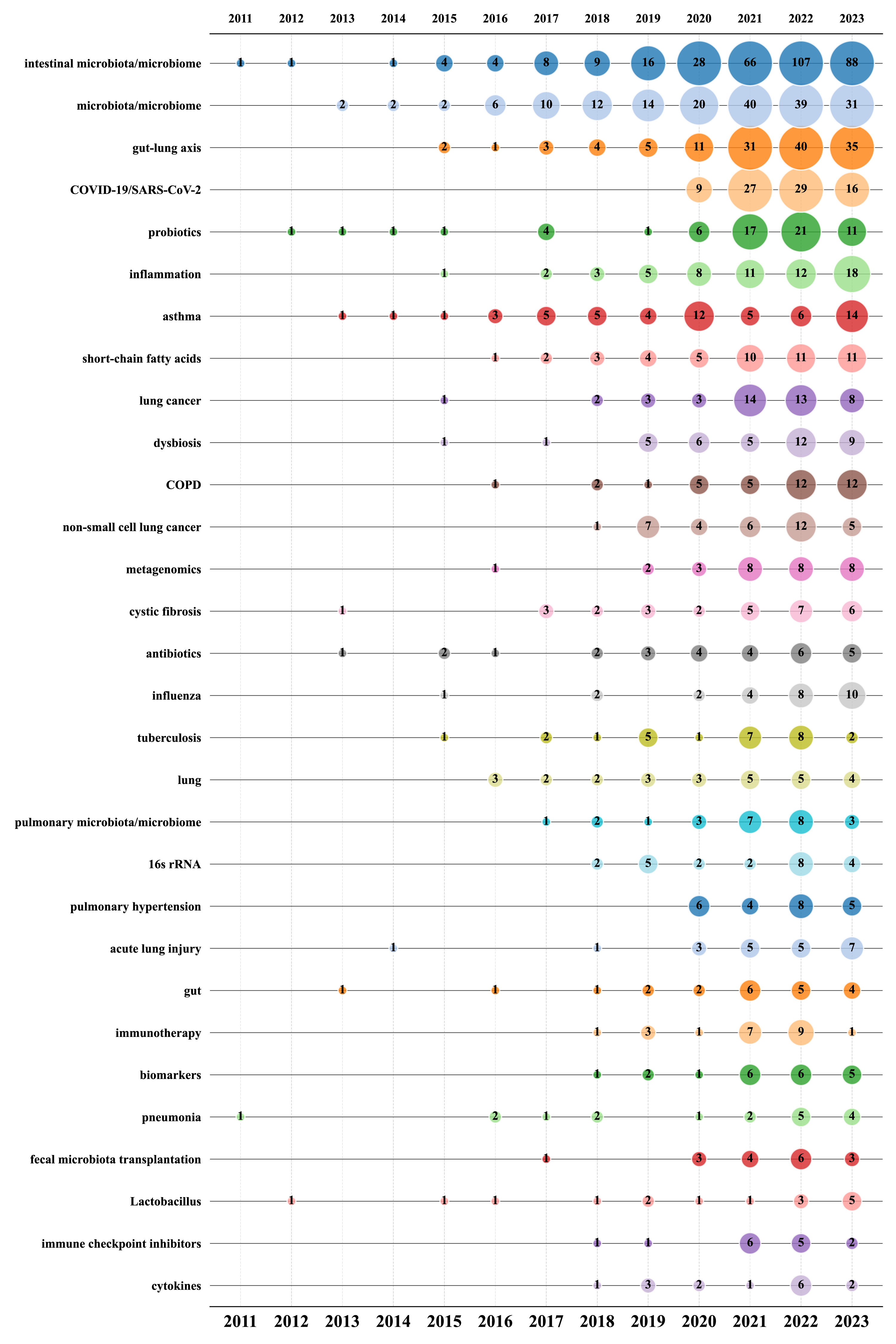

Supplement: Supplementary Figure 3 — Bubble chart of the top 30 author keywords by year. [file Image_3.tif]
